# The prognosis of women with stage IB1-IIB node-positive cervical carcinoma after radical surgery

**DOI:** 10.1186/1477-7819-2-47

**Published:** 2004-12-18

**Authors:** Xi Cheng, Shumo Cai, Ziting Li, Meiqin Tang, Muquan Xue, Rongyu Zang

**Affiliations:** 1 Department of Gynecologic Oncology, Cancer Hospital, Fudan University, Shanghai, 200032, P.R. China

## Abstract

**Background:**

Pelvic lymph nodes metastasis is an important prognostic factor for patients with cervical carcinoma. However, the relationships between the number of positive nodes, site of metastases nodes, adjuvant therapy and the prognosis is controversial. The purpose of this study was to investigate the influence of positive lymph nodes on the prognosis of Chinese women with stage IB1-IIB cervical carcinoma.

**Patients and methods:**

Between January 1992 and December 1997, 398 women with International Federation of Gynecology and Obstetrics (FIGO) stage IB1-IIB cervical carcinoma underwent radical surgery in Cancer Hospital, Fudan University. Of these sixty-six patients (16.6%) who were histologically confirmed to have positive pelvic lymph nodes were analyzed retrospectively. The survival was estimated using Kaplan-Meier method. The differences in survival were compared with Log-rank test. Multivariate analyses were performed with the Cox proportional hazard model.

**Results:**

The 5-year survival of the patients with pelvic lymph nodes metastases was 40.7%. Cox proportional hazard model analysis showed that cellular differentiation, the number of positive nodes and adjuvant therapy to be the independent prognostic factors (*P *< 0.05). The 5-year survival of patients with one positive node was higher than that of those with two or more positive nodes (56.5% vs. 36.4%, *P *< 0.05). The distant metastasis rate in the former group (5.9%) was lower than the latter's (32.7%) (*P *= 0.05). However, there was no significant difference of pelvic recurrence between the two groups (*P *> 0.05). The number of positive nodes positively correlated with the level of positive nodes (*P *< 0.01). The 5-year survival of the patients who had no adjuvant therapy (12.6%) was much lower than that (53.7%) of those with adjuvant therapy (*P *< 0.05). However, there was no obvious difference between adjuvant radiotherapy, chemotherapy and chemo-radiotherapy (*P *> 0.05).

**Conclusions:**

The prognosis of patients with stage IB1-IIB node-positive cervical carcinoma who underwent radical surgery alone was very poor. Adjuvant therapy increases the survival rate, decreases the pelvic recurrence and distant metastasis.

## Background

Although radical radiotherapy (RT) and radical surgery can be the proper choices for patients with early stage cervical cancer, most of the patients in China prefer the radical surgery to RT. Hence, in China the radical surgery has been widely used as first-line therapy for this group of women. Some poor prognostic subgroups have been identified, among these the pelvic lymph node status has been considered as the most important prognostic factor. Radical hysterectomy with bilateral pelvic lymphadenectomy produces an expected 85–90% survival in women with stage IB and IIA cervical carcinoma without lymphatic spread. However, once tumors involve regional lymph nodes, 5-year survival has been reported to be only 30–60% [1]. In most of the studies the presence of pelvic lymph node metastases has been associated with increased pelvic recurrence and distance metastases, and a decrease in overall survival [2-7]. However, many questions such as the relationship between the numbers, the site of positive nodes, the modality of postoperative multidisciplinary therapy and the prognosis is not yet clear. This study investigated the factors that could predict the prognosis of the patients with stage IB1-IIB node-positive cervical carcinoma.

## Patients and methods

Between January 1992 and December 1997, 398 women with International Federation of Gynecology and Obstetrics (FIGO) stage IB1-IIB cervical carcinoma underwent radical surgery at the Department of Gynecologic Oncology, Cancer Hospital of Fudan University. Of these 66 patients who had undergone Wertheims-Meigs' surgery (radical hysterectomy and pelvic lymphadenectomy) and were histologically confirmed to harbor positive pelvic lymph node were included in this study. The median age at diagnosis was 49 years (range 21 to71). Out of 66, 8 patients were in stage IB1 (12.1%), 37 patients (56.1%) in stage IIA and 21 patients (31.8%) in stage IIB. Histologically 41 women (62.1%) had squamous carcinoma, 20 (30.3%) had adenocarcinoma, 4 (6.1%) adenosquamous carcinoma and 1 patient (1.5%) had small cell carcinoma. The tumors in 4 patients (6.1%) were well differentiation, 46 cases (69.7%) moderately differentiated and 16 (24.2%) poorly differentiated. The average lymph nodes resected were 14.8 per patient while the average positive lymph nodes resected were 3.7 (1~28) per patient. The average diameter of the cervical tumors was 3.6 cm (1~7 cm). The details of the patients' clinical characteristics are listed in Table 1.

**Table 1:** Clinico-pathologic characteristics of patients with node-positive cervical carcinoma after radical surgery

**Factors**	**n**	**Percentage (%)**
Age(yrs)		
<40	15	22.7
≥40	51	77.3
Stage		
IB	8	12.1
IIA	37	56.1
IIB	21	31.8
Tumor size(cm)		
<4	31	47.0
≥4	35	53.0
Histology		
Squamous	41	62.1
Adenocarcinoma	20	30.3
Adenosquamous	4	6.1
Others	1	1.5
Differentiation		
Poor	16	24.2
Moderate	46	69.7
Well	4	6.1
Pelvic lymph node metastases		
1	17	25.8
≥2	49	74.2
Parametrial extension		
Negative	58	87.9
Positive	8	12.1
Vaginal margin involved		
Negative	64	97.0
Positive	2	3.0
Depth of stromal invasion		
≤2/3	19	28.8
≤2/3	47	71.2
Lymphvascular permeation		
Negative	47	71.2
Positive	19	28.8

Sixty four of these women (97.0%) had brachytherapy in either three or four fractions with a total dose of 15~20 Gy at point A, two weeks prior to radical surgery because of bulky tumor or vaginal vault involvement. Intra-arterial chemotherapy was administered to 11 patients (16.7%) before surgery because of bulky tumor or parametrial extension. The regimen based on cisplatin (CDDP) + 5-Fluorouracil(5-FU) with 2~3 cycles at 3-weeks intervals was used. All patients underwent Wertheims-Meigs' procedure that included radical hysterectomy and bilateral pelvic lymphadenectomy. Three patients had para-aortic lymph node (PALN) sampling because there was suspicion of metastasis.

Postoperative external beam irradiation was delivered to 18 patients (27.3%) with 6 MV X-ray routine anterior and posterior portal at a dose of 35~45 Gy at point B with 1.8 Gy daily fraction. Two patients with positive vaginal margin were given extra brachytherapy with 15 Gy 0.5 cm below the vaginal mucosa. 12 cases with positive common iliac node and 3 with PALN metastasis received an additional 40 Gy to the PALN chain area. 10 patients (15.2%) were given postoperative chemotherapy. The regimen consisted CDDP + 5-FU + Cyclophosphamide (CTX) for squamous carcinoma, and CDDP + 5-FU + Mitomycin (MMC) for adenocarcinoma with 2~6 cycles at 3~4-weeks intervals. A total of 19 patients (28.8%) received adjuvant radiotherapy and chemotherapy as mentioned above.

### Follow-up

Patients were evaluated every two months for the first two years and then six monthly for the additional years by clinical interview, or telephone, or letters. Disease-free survival (DFS) was defined as the time from the date of surgery to local or nodal recurrence or metastasis. Overall survival (OS) was calculated from the date of surgery to the date of death. Recurrences were defined as local if they were detected in the pelvis or vagina and distant metastases as detected in extra-pelvic locations. The median follow-up time was 32 months (range 2~108 months).

### Statistical analysis

Data analysis was performed with Statistical package for social sciences (SPSS) version 10.0 statistical package. The survival was calculated by Kaplan-Meier method. The differences in survival were compared with Log-rank test. Multivariate analyses were performed with the Cox proportional hazard model. The correlation analysis was performed by Kendall's method. Pearson's chi-square or Fisher's exact test was used to compare the difference of proportions. A probability value of *P *< 0.05 was considered significant.

## Results

**Figure 1:**
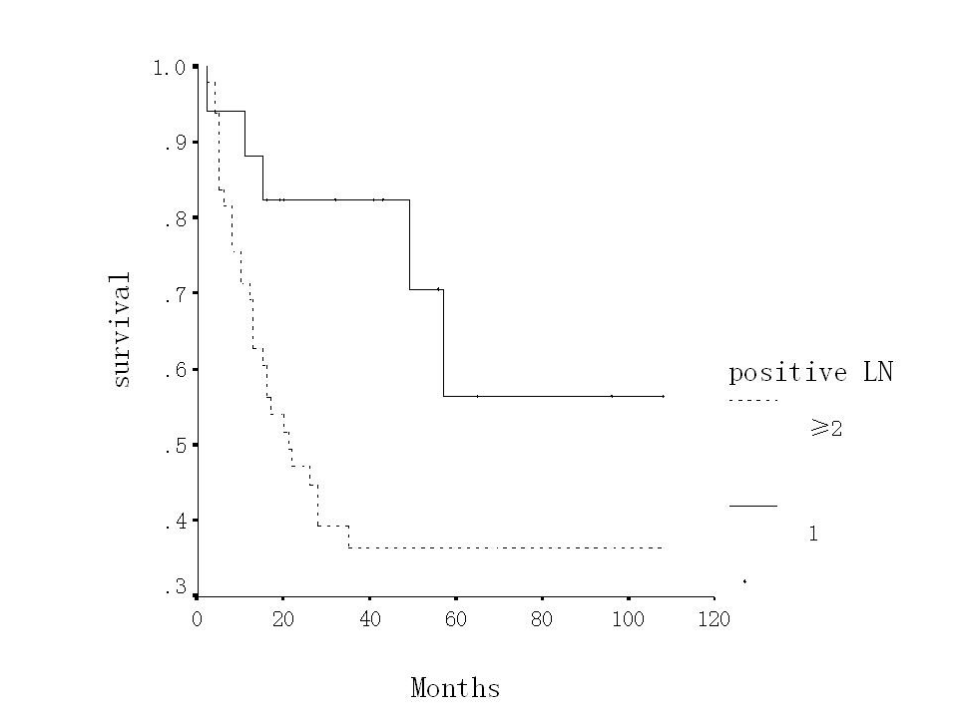
Overall survival according to one vs. two or more positive lymph nodes.

The 5-year overall survival of the patients with pelvic lymph node metastasis was 40.7%. The 5-year survival of patients with one positive node (56.5%) was higher than that (36.4%) of those with two or more positive nodes (*P *< 0.05). The former's distant metastasis rate (5.9%) was lower than the latter's (32.7%) (*P *= 0.05). However, there was no significant difference of pelvic recurrence between them (*P *> 0.05) (Table 2, Figure 1)

**Table 2:** Relationship between the number of positive nodes and prognosis

**Number of positive nodes**	**n**	**5-year survival (%)**	***P***	**Recurrence rate (%)**	***P***	**Metastasis rate (%)**	***P***
1	17	56.5	0.033	23.5	0.807	5.9	0.050
≥2	49	36.4		26.5		32.7	

The 5-year survival (33.3%) of the patients with positive common iliac node and paraaortic lymph node (PALN) was less than that (43.1%) of   patients with lower than common iliac node involvement. However, the difference was not significant (P > 0.05). The former's pelvic recurrence rate (13.3%) was lower than the latter's (29.4%) (P > 0.05). However, the patients with common iliac or paraaortic nodes had significantly higher distant metastasis (53.3%) than that of the patients with lower nodes (17.6%) (P < 0.01). The number of the positive lymph nodes was closely correlated with the level of lymph node metastasis. The relative coefficient was 0.557 (P < 0.01) (Table 3).

**Table 3:** Relationship between the site of positive nodes and prognosis

**Site**	**n**	**5-year survival rate (%)**	**P**	**Recurrence rate (%)**	**P**	**Metastasis rate (%)**	**P**
Common iliac or above	15	33.3	0.086	13.3	0.318	53.3	0.005
Lower than common iliac	51	43.1		29.4		17.6	

**Figure 2:**
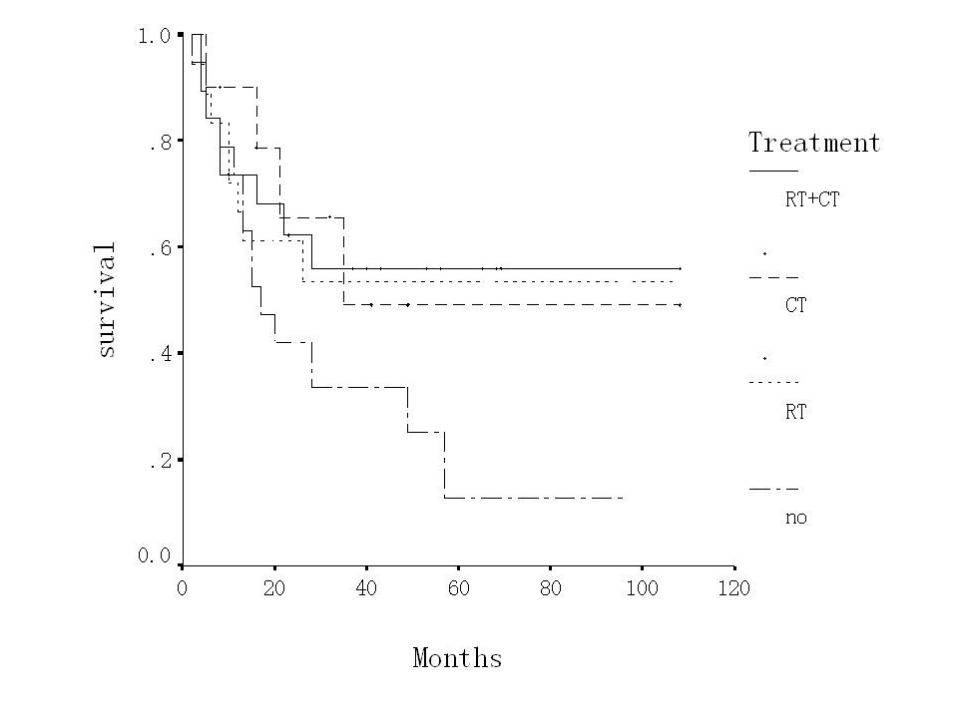
Overall survival according to adjuvant therapy [radiotherapy (RT) vs. chemotherapy (CT) vs. radiochemotherapy (RT+CT) vs. no adjuvant therapy (No)].

The 5-year survival (12.6%) of the   patients who had no adjuvant therapy was much lower than that (53.7%) of   those with adjuvant therapy (P<0.05). The 5-year survival rates of the adjuvant radiotherapy, adjuvant chemotherapy, and adjuvant radio-chemotherapy groups were 53.5%, 49.2% and 56.1% respectively (P > 0.05). The pelvic recurrence (42.1%) and distant metastasis (31.6%) of the surgery alone were higher than those with adjuvant therapeutic groups. However, the differences were not significant (P > 0.05) (Table 4, Figure 2)

**Table 4:** Relationship between adjuvant therapy and prognosis

	**n**	**5-year survival (%)**	**Recurrence rate (%)**	**Metastasis rate (%)**
No adjuvant therapy	19	12.6	42.1	31.6
Radiotherapy	18	53.5	22.2	22.2
Chemotherapy	10	49.2	20.0	10.0
Radiochemotherapy	19	56.1	15.8	21.1

### Multivariate analysis of prognostic factors

Cox proportional hazard model analysis showed that cellular differentiation, number of positive nodes and adjuvant therapy to be the independent prognostic factors for survival (*P *< 0.05) (Table 5)

**Table 5:** Cox proportional hazard model analysis of variables in predicting overall survival

Factors	Coefficient	RR	95%CI	P
Age	-0.031	0.970	0.932~1.010	0.136
Stage	0.283	1.327	0.379~4.644	0.658
Tumor size	0.332	1.394	0.984~1.975	0.061
Histology	-0.035	0.966	0.563~1.659	0.900
Differentiation	0.787	2.196	1.104~4.370	0.025*
Number of positive nodes	0.076	1.079	1.006~1.158	0.034*
Parametrial extension	0.484	1.623	0.584~4.508	0.353
Vaginal margin involved	-1.007	0.365	0.029~4.655	0.438
Depth of stromal invasion	0.423	1.526	0.856~2.722	0.152
Lymphvascular permeation	0.270	1.311	0.500~3.437	0.582
Nerve invasion	0.101	1.106	0.092~13.337	0.937
Adjuvant therapy	-1.684	0.186	0.075~0.459	0.000*

* *P *< 0.05

## Discussion

Cervical cancer remains the third common cancer in women around the world, although its incidence is on the decline in North American and in Europe. It is estimated that there will be 10,370 new cases in 2005 in North America [8]. In many developing countries, not only is cervical cancer the most frequently occurring cancer among middle-aged women, but it is also a leading cause of death, partly due to the poor access to medical care and the unavailability of routine screening in many of these countries [9]. In Shanghai, the biggest city in China, there were 150 new cases in 2000. The standard incidence rate was 2.9 per 100 000 [10].

Radical surgery has been found to be very effective in patients with early stage (IB-IIA) cervical carcinoma [11-13]. However, the 5-year survival has been lingering at 50%-90% during the past twenty years [14]. The reported risk factors of cervical carcinoma included clinical stage, bulky tumor, histological types, cellular differentiation, deep stromal invasion, parametrial extension, vaginal margin involved, lymphvascular permeation, lymph node metastasis, race and age [13,15-18]. In most studies the presence of pelvic lymph node metastases has been associated with increased pelvic recurrence and distance metastases, and a decrease in overall survival. Our Cox proportional hazard model analysis showed cellular differentiation, number of positive nodes and adjuvant therapy to be the independent prognostic factors (*P *< 0.05). The 5-year survival of patients with one positive node (56.5%) was higher than that (36.4%) of those with two or more positive nodes (*P *< 0.05). There was no significant difference of pelvic recurrence between them. However, the former's distant metastases rate (5.9%) was lower than the latter's (32.7%) (*P *= 0.05). In the analysis of the site of lymph node metastases, the 5-year survival (33.3%) of the patients with positive common iliac node and paraaortic lymph node (PALN) was lower than that (43.1%) of patients with lower than common iliac node involvement. Unlike the series of Tsai the difference in our series was not statistically significant [19]. This is probably due to limited number of cases with paraaortic or common iliac nodes. The number of positive nodes correlated with the height of positive nodes (*P *< 0.01). The result suggested that in patients with more positive pelvic nodes there is higher chance of nodal metastasis at higher level node basins, which supported the external irradiation of PALN chain area for the multiple pelvic nodes involvement. The distant metastasis rate was 32.7% in patients with multiple positive lymph nodes, which showed the limitation of adjuvant radiotherapy and theoretically supported the postoperative combined chemotherapy.

The prognosis of patients with positive lymph node was poor because of local recurrence and distant metastasis. How to improve the prognosis of these patients has been the focus of gynecological oncology. Stock *et al *[7] compared postoperative whole pelvic irradiation with those treated with radical hysterectomy alone, and showed a significantly improved pelvic control rate, 5-year disease-free survival (DFS) and overall survival (OS) (78% Vs 45%, 65% Vs 41%, 58% Vs 46%, respectively).

Peters' study [20] demonstrated that postoperative radio-chemotherapy could greatly improve the 4-year DFS and OS compared to the adjuvant radiotherapy alone. In our study, adjuvant therapy improved the 5-year survival than the surgery alone (53.7% vs. 12.6%), decreased the pelvic recurrence and distant metastasis, which suggested the clinical importance of adjuvant therapy in patients with positive nodes. In this study the 5-year survival of adjuvant radio-chemotherapy arm was higher than adjuvant radiotherapy alone or chemotherapy alone. The pelvic recurrence (42.1%) and distant metastasis (31.6%) of the surgery alone were higher than the other three therapeutic arms. However, the differences were not significant and the limited number of cases in each arm could be the reason. It is necessary that these results are verified in prospective randomized control trials.

Several randomized clinical trials [20-25] performed by the Gynecologic Oncology Group (GOG), the Radiation Therapy Oncology Group (RTOG) and the Southwest Oncology Group (SWOG) have demonstrated a significant advantage both in DFS and OS when cisplatin-based chemotherapy was administered during radiation for advanced stages of cervical cancer and early stage disease with poor prognostic factors. Green *et al *[26] did a systemic review of all known randomized controlled trials published between 1981 and 2000, 2865–3611 patients were available for analysis. The findings suggested that concomitant chemotherapy (CT) and radiotherapy (RT) improves OS and DFS, and reduces local and distant recurrence. Based on results, the US National Cancer Institute (NCI) released a clinical announcement supporting the concurrent use of cisplatin-based chemotherapy with RT for high-risk early stage and locally advanced stage cervical cancer [9,27]. Recently a prospective randomized trial performed by the National Cancer Institute (NCI) of Canada [25] failed to show benefit with the use of chemoradiation compared with RT alone. The potential inclusion of paraaortic nodes positive patients and the significant difference in anemia raises question about this trial result [28].

The therapy of patients with stage IIB cervical carcinoma is still a controversy [29]. Although radical radiotherapy (RT) is the proper choice for patients with stage IIB cervical cancer in general, the therapeutic effect is not as good as expected if the tumor is too bulky or is histologically adenocarcinoma. Hence a few gynecology oncologists tried brachytherapy and/or neoadjuvant chemotherapy to decrease the lesion, then performed radical surgery for some stage IIB patients with bulky tumor (>4 cm) or slight (less than 1/2) parametrial extension [30,31]. Postoperative adjuvant therapy would then be recommended according to risk factors [9,17,20,32]. In our study, the 5-year survival of the patients with stage IIB cervical carcinoma was only 54.3%, which was not at all satisfactory compared with the reported 58.9%–80.1% [33-35]. So we advocate that radical surgery should be taken cautiously for this group of patients. Any attempt to improve their prognosis by means of adjuvant therapy is not recommended if the parametrium can not be thoroughly dissected from the pelvic wall.

## Competing interests

The author(s) declare that they have no competing interests.

## Authors Contributions

**XC **collected the data, patients' follow-up and drafted the article.

**SC, ZL, MT, MX **collectively designed the study, participated in collection of data and revising the article.

**RZ **participated in collection and analysis of data, revising the article

## Funding Source

None
